# Familial Epilepsy Associated With Concurrent CHRNB2 Mutation and RBFOX1 Exon Deletion: A Case Report

**DOI:** 10.7759/cureus.35845

**Published:** 2023-03-06

**Authors:** Tony Xu, Dorris Luong, Ning Zhong

**Affiliations:** 1 Math Science Technology, Paul Laurence Dunbar High School, Lexington, USA; 2 Neurology, Kaiser Permanente Sacramento Medical Center, Sacramento, USA; 3 Epilepsy, North Valley Comprehensive Epilepsy Program, Kaiser Permanente Sacramento Medical Center, Sacramento, USA

**Keywords:** rbfox1, chrnb2, variants of uncertain significance, epilepsy, focal onset seizure

## Abstract

Understanding the genetic basis of epilepsy may lead to an improved understanding of its etiology, more precise medical management, and ultimately improved outcomes. It is imperative for patients with epilepsy to obtain a molecular diagnosis, especially when strong familial epilepsy is discovered. We investigated a multi-generational family with epilepsy. The proband was a 19-year-old female who experienced focal onset seizures, with presenting symptoms of feeling dizzy, disorientation, and loss of consciousness. Her electroencephalography (EEG) studies revealed interictal focal slowing and sharp waves in both the left or right hemispheres independently. EEG monitoring showed that the seizures arose from the left fronto-temporal region and her brain MRI was normal. The proband's sister also suffered from focal onset seizures. Her EEG showed focal epileptiform discharge in the right temporal region, and her brain MRI was unrevealing. Two genetic tests were conducted for the proband: 1) array comparative genomic hybridization (CGH) revealed 16p13.3 deletion but no 22q deletion; and 2) next generation sequencing (NGS) Epilepsy Panel revealed a few variants of uncertain significance (VUS), including in CHRNB2 (c.1423A>G, p.Ile475Val) and RBFOX1 (RNA binding fox-1 homolog 1) (exon1-2 deletion). The proband’s sister also carries both the CHRNB2 (cholinergic receptor nicotinic beta 2) variant and RBFOX1 deletion. The proband’s father carries the CHRNB2 variant, and her brother and mother carry the deletion of RBFOX1. In this family, the co-expression of the CHRNB2 variant and RBFOX1 deletion may cause the clinical seizures seen in the proband and her sister. It is also possible that the RBFOX1 deletion is associated with an increased risk of seizure disorder with variable expressivity.

## Introduction

Epilepsy, a common neurological disorder affecting over 50 million people worldwide, exhibits high heterogeneity by clinical presentation, causative etiologies, and triggering factors among others. Finding a treatable cause of epilepsy is the foundation of precision medicine, which considers individual patient characteristics and offers customized treatment for patients with epilepsy [1, 2]. With advances in genetic sequencing and variant/mutation detecting technology, more and more epilepsy cases are studied. It was estimated that in 70% of epilepsy cases, genetic factors are responsible either as a single genetic variant in rare epilepsies (epilepsy syndromes) or as multiple genetic variants acting along with different environmental factors in common epilepsies [3, 4, 5]. Understanding the genetic basis of epilepsy may lead to an improved understanding of the etiology of epilepsy, precision medical management, and ultimately, improved outcomes [6]. It is imperative for patients with epilepsy to obtain a molecular diagnosis as early as possible, especially when a strong familial history is discovered [6].

To date, different approaches have been applied to explore the genetic factors in epileptogenesis and to discover new treatment targets [4]. In specific epilepsy syndromes, mutilations in a series of genes have been identified as the core cause of epilepsy, such as SCN1A mutations associated with febrile seizure plus syndrome, whereas some genes may be associated with brain structural lesions which cause epilepsy, such as TSC genes in tuberous sclerosis, and KIRT1 and CCM2 genes in cavernous malformations) [4]. Due to the heterogeneous phenotypic features of epilepsy involved in changes in the sequence and expression of multiple genes (instead of a single gene), as well as the modification and regulation of genes by multiple factors, gene co-expression network analysis may help to reveal the molecular regulatory mechanisms during epileptogenesis [7]. The utility of genetic testing in peripheral blood samples from patients with epilepsy remains less explored, especially the discovery of variants of uncertain significance (VUS), which often leaves challenges for epileptologists’ clinical practice [8].

Previously variants in CHRNB2 (cholinergic receptor nicotinic beta 2) have been studied and linked to autosomal dominant nocturnal frontal lobe epilepsy [9]. Partial deletions of the RBFOX1 (RNA binding fox-1 homolog 1) gene encoding the neuronal splicing regulator have been reported in a wide range of neurodevelopmental and neuropsychiatric disorders [10]. Our case report describes the discovery of VUS of the CHRNB2 and RBFOX1 genes in a multi-generation family with epilepsy.

The article was previously posted to the Research Square preprint server on February 03, 2023 (https://doi.org/10.21203/rs.3.rs-2493257/v1). 

## Case presentation

Case presentation

A three-generation family was studied. The proband and her sister were studied with electroclinical and radiological phenotyping which incorporated EEG, brain imaging, and molecular genetic analysis. Medical information and genetic tests were obtained from the other family members.

Clinical characteristics

The proband was a 19-year-old female with her first episodes manifested as collapse while dancing. Thereafter, she continued to experience episodes of feeling dizzy, and disoriented, followed by loss of consciousness, episodes lasting 30-60 seconds. She was amnestic of some of her spells. Cardiac workup with event monitoring and a stress test were unrevealing. Her brain MRI did not reveal a lesion. To further characterize her episodes, she underwent video EEG (vEEG) monitoring. Interictally, focal slowing and sharp waves were seen in the left or the right centro-parietal regions or the right frontocentral regions independently. One focal onset non-impaired awareness seizure was reported and recorded. Clinically the patient felt dizzy and lightheaded and experienced palpitations. She was briefly confused and did not know where she was. Afterward, she was able to recall what happened and to describe the event to her family and providers.

**Figure 1:**
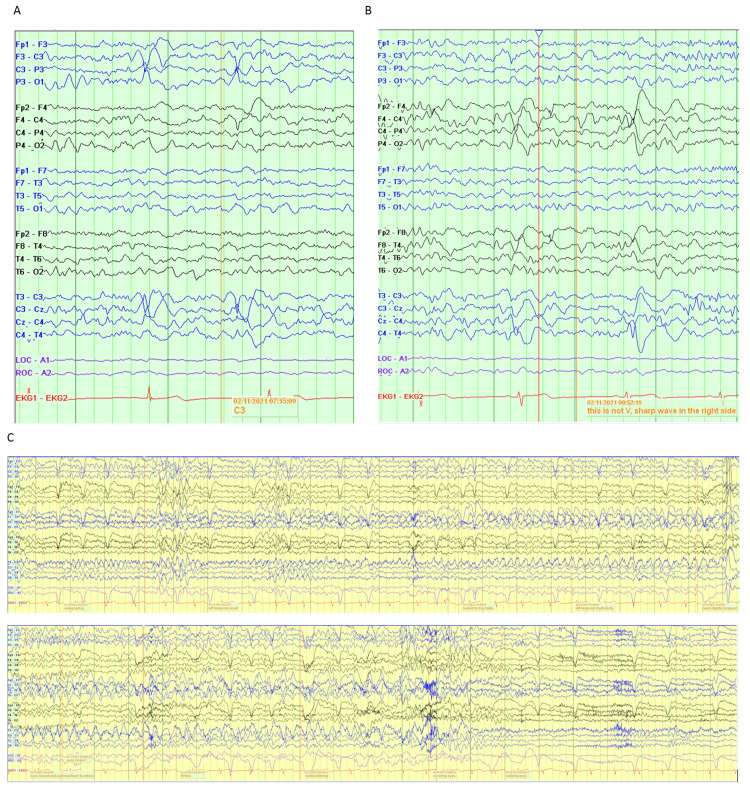
vEEG data A) Sharp waves distributed in the left centroparietal areas, shown in the longitudinal bipolar (double banana montage. B) Sharp waves were seen in the right frontocentral regions or right centroparietal regions, shown in the longitudinal bipolar montage. C) Ictal EEG showed low voltage 4-5 Hz rhythmicity was seen in the left temporal region, followed by higher voltage 3-4 Hz rhythmic delta activity with sharply contoured morphology in the same region. vEEG: video electroencephalography

On review of the video recording, the patient was observed laying supine with eyes blinking during the episode. Subsequently, she sat up and rubbed her eyes at the end of the ictal pattern. Electroencephalography (EEG) showed the ictal pattern started as low voltage 4-5 Hz rhythmicity in the left frontotemporal region maximum at T3, followed by modest voltage amplitude 3-4 Hz rhythmic delta activity with sharply contoured morphology in the same regions. The frequency then slowed to 3 Hz with an even higher voltage. The ictal pattern ended with irregular delta activity in the left hemisphere followed by normal awake background recording. The seizure pattern lasted for 30 seconds. Ictally, the heart rate transiently increased from a baseline of 54 bpm to 73 bpm (Figure 1).

**Figure 2:**
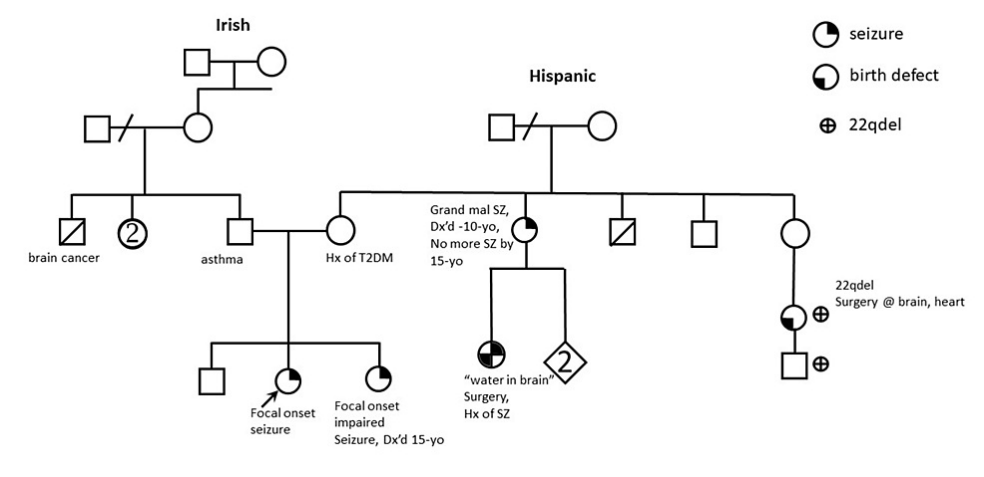
Family pedigree Hx: history; SZ: seizure; Dx'd: diagnosed; T2DM: type 2 diabetes mellitus; yo: years old

Based on the vEEG morning data, it was confirmed that the proband suffers from focal onset seizures. Upon further exploration of her family history, it was found that the proband's sister had focal onset seizures since adolescence, and the proband’s maternal aunt and maternal cousin also had childhood-onset seizures presenting with a mixture of partial and generalized seizures (Figure 2).

The proband’s sister had her first event at 15 years old. Thereafter, she continued experiencing focal onset seizures with or without impaired awareness (often clustering seizures) despite treatment with different anti-seizure medications (ASMs). The brain MRI was unrevealing. Her EEG demonstrated focal epileptiform discharges in either the left or right temporal regions.

Genetic testing

Due to a strong family history of epilepsy, we extended the workup to genetic tests. The following genetic test results were discovered in the proband: 1) array CGH (comparative genomic hybridization) revealed 16p13.3 deletion but no 22q deletion, and 2) NGS (next generation sequencing) Epilepsy Panel revealed a few VUS: CHRNB2 (c.1423A>G, p.Ile475Val), RBFOX1 (exon1-2 deletion), and SYNJ1 (Table 1). The discovered genetic variants have not been previously reported in the scientific literature or in the HGMD (Human Gene Mutation Database) databases, though the genes were reported to be associated with epilepsy. 

**Table 1:** Identified VUS in the proband VUS: variants of uncertain significance; UID: Unique Identifier; PMID: PubMed Identifier

Gene	Variant	Zygosity	Reported association with epilepsy
CHRNB2	c.1423A>G (p.Ile375Val)	Heterozygous	Autosomal dominant nocturnal frontal lobe epilepsy (MedGen UID: 344263)
RBFOX1	Deletion (Exons 1 – 2)	Heterozygous	Autosomal dominant idiopathic generalized epilepsy (PMID: 23350840, 24039908, 25950944, 26174448)
SYNJ1	c.2230A>G (p.Ile744Val)	Heterozygous	Autosomal recessive developmental and epileptic encephalopathy, early infantile epileptic encephalopathy (MedGen UID: 1374886)

With help from medical genetics consultation, genetic tests were obtained from the proband’s immediate family members. The proband’s sister also carries both the CHRNB2 variant and the RBFOX1 deletion. Family studies confirmed the 16p13.3 deletion (including the RBFOX1 deletion) was maternally inherited and the CHRNB2 VUS was paternally inherited; the proband’s father carries the CHRNB2 variant, and the proband’s mother and brother carry the deletion of RBFOX1. None of the carriers (the proband's father, mother, and brother) have seizures. Another maternal cousin and her son were discovered to have the 22q deletion syndrome with birth organ defects - such information was thought to be irrelevant to the proband and her sister’s epilepsy. The maternal aunt and cousin who have seizures were not tested, as additional family studies were unlikely to add additional information given the revealed inheriting pattern.

VUS protein structure analysis

**Figure 3:**
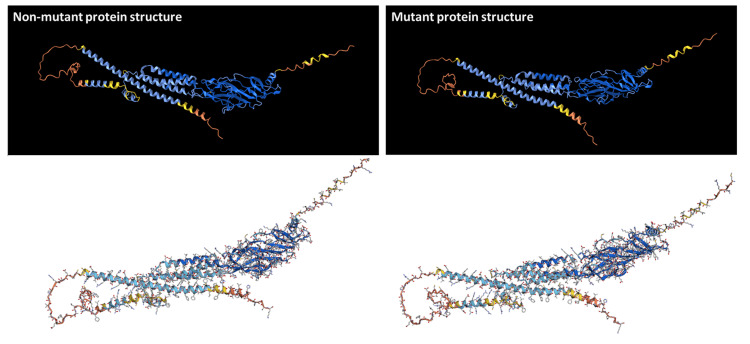
Predicting the impact of CHRNB2 c.1423A>G variant on the protein structure

Since the patient's father is seizure-free, it is unclear if the CHRNB2 VUS is associated with a significant risk for seizures. We applied AlphaFold AI (artificial intelligence) and deep learning to investigate if the discovered CHRNB2 gene mutation affects the protein structure. It revealed that the discovered CHRNB2 VUS is not likely to impact the protein structure (Figure 3), which strongly suggests that such VUS is unlikely to cause familial epilepsy.

## Discussion

CHRNB2 is responsible for the clinical phenotype in some Autosomal Dominant Nocturnal Frontal Lobe Epilepsy (ADNFLE) patients [9]. The electroclinical studies from the proband and her sister did not provide evidence that their seizures were frontal lobe epilepsy. Instead, both were likely suffering from temporal lobe epilepsy. As we predicted, the discovered CHRNB2 VUS was not likely to impact the protein structure - indicative of the fact that CHRNB2 VUS is unlikely to be pathogenic.

RBFOX1 is a neuron-specific splicing factor that exerts both positive and negative regulatory effects on alternative splicing and is associated with several neurodevelopmental and neuropsychiatric disorders including autism spectrum disorder (ASD), developmental delay, epilepsy, ADHD (attention-deficit/hyperactivity disorder), and psychiatric disorders [10, 11]. Within our reported family, it is interesting to note that both sisters and the carriers (proband’s mother and brother) with the discovered RBFOX1 deletion variant do not present with any neuropsychiatric clinical manifestation. Such observation raised the possibility that in this family, the RBFOX1 deletion may be associated with an increased risk of seizure disorder with variable expressivity.

It is interesting to note that the proband and her sister are the only family members with reported seizures, and they carry both VUS in CHRNB2 and the RBFOX1 deletion. We further explored the possibility of co-expression of VUS in CHRNB2 and the RBFOX1 deletion affecting the phenotype in the proband and her sister.

A recent gene ontology analysis revealed that genes with decreased expression, affected by RBFOX1, are involved in neuronal development, synapse function, and cell differentiation (Table 2). Within the gene members identified, CHRNB2 was noted within the expression network affecting neurogenesis, neuronal development, and synaptic transmission [10]. The co-expression of both the CHRNB2 variant and RBFOX1 deletion may likely have caused the clinical seizures seen in the proband and her sister.

**Table 2:** Gene ontologies of RBFOX1-dependent differentially spliced genes GO: gene ontology

GO term	P-value	Gene members
Genes with decreased expressions		
GO:0022008 - neurogenesis	3.37 × 10^-9^	GPRIN1, NRTN, CDK5R1, HELT, NNAT, ONECUT2, TH, L1CAM, BRSK1, KIT, KCNIP2, GPC2, BCL11B, MAPT, BAI1 ROBO2, DCX, DNMT3B, TUBB3, DSCAM, DTX1, NRXN3, STMN2, SOX11, CELSR3, ARTN, NRXN1, DLX2, DLX1, SEMA6C, DLX5, PDGFRA, CNTN2, CHRNB2, KALRN
GO:0048666 - neuron development	1.41 × 10^-5^	GPRIN1, CDK5R1, NRTN, NRXN3, ONECUT2, TH, CELSR3, L1CAM, NRXN1, KCNIP2, SEMA6C, DLX5, BCL11B, BAI1, CNTN2, ROBO2, CHRNB2, DCX, DSCAM, KALRN
GO:0032990 - cell part morphogenesis	7.01 × 10^−5^	CDK5R1, NRXN3, ONECUT2, CELSR3, L1CAM, NRXN1, SEMA6C, DLX5, BCL11B, BAI1, CNTN2, CHRNB2, ROBO2, DCX, DSCAM, KALRN
GO:0007268 - synaptic transmission	1.13 × 10^−4^	KCNMB4, GABRB3, NRXN3, SLC12A5, TH, BSN, NRXN1, KCNIP2, GAD2, GRIA2, SYN1, DLG4, CHRNB2, CORT, KCNQ2, GAD1, CACNA1B
GO:0000902 - cell morphogenesis	2.82 × 10^−4^	ARHGEF2, CDK5R1, NRXN3, ONECUT2, CELSR3, L1CAM, NRXN1, BRSK1, SEMA6C, DLX5, BCL11B, BAI1, CNTN2, ROBO2, CHRNB2, DCX, DSCAM, KALRN
GO:0007417 - central nervous system development	7.83 × 10^−4^	CDK5R1, HELT, MAFB, SOX11, SLC6A3, NNAT, BCAN, CYP26A1, ZBTB16, TAGLN3, DLX2, DLX1, HOXB2, CHD7, SBK1, BCL11B, ROBO2, CHRNB2, DCX

The precise causative mechanism of co-expression between RBFOX1 deletion and the VUS in CHRNB2 is still uncertain. Further research is encouraged to confirm the discovered variants and other mutations as possible epilepsy pathogenic in the larger population. Our observation and analysis highlight the need for increasing awareness of and access to genetic testing for adult epilepsy patients with a strong epileptic family history. The VUS are so rare that there are no previous reports. Based on family studies and genome-wide association studies, a fair number of VUS might be reclassified [12].

## Conclusions

Genetic findings will provide valuable insight into the natural history of epilepsy heterogeneity. In recent years, the awareness of the impact of genetic background on developing drug-resistant epilepsy has increased, and new genetic techniques are proving very useful in clinical practice, which may help clinicians to explain the clinical presentation of drug-resistant epilepsy. In addition, this will likely help us understand the impact of personalized and precise pharmacological treatment on epilepsy.
